# A Bowman-Birk type chymotrypsin inhibitor peptide from the amphibian, *Hylarana erythraea*

**DOI:** 10.1038/s41598-018-24206-4

**Published:** 2018-04-11

**Authors:** Luyao Zhang, Xiaoling Chen, Yue Wu, Mei Zhou, Chengbang Ma, Xinping Xi, Tianbao Chen, Brian Walker, Chris Shaw, Lei Wang

**Affiliations:** Natural Drug Discovery Group, School of Pharmacy, Queen’s University, Belfast, BT9 7BL Northern Ireland UK

## Abstract

The first amphibian skin secretion-derived Bowman-Birk type chymotrypsin inhibitor is described here from the Asian green frog, *Hylarana erythraea*, and was identified by use of molecular cloning and tandem mass spectrometric amino acid sequencing. It was named *Hylarana erythraea* chymotrypsin inhibitor (HECI) and in addition to inhibition of chymotrypsin (Ki = 3.92 ± 0.35 μM), the peptide also inhibited the 20 S proteasome (Ki = 8.55 ± 1.84  μM). Additionally, an analogue of HECI, named K^9^-HECI, in which Phe^9^ was substituted by Lys^9^ at the P1 position, was functional as a trypsin inhibitor. Both peptides exhibited anti-proliferation activity against the human cancer cell lines, H157, PC-3 and MCF-7, up to a concentration of 1 mM and possessed a low degree of cytotoxicity on normal cells, HMEC-1. However, HECI exhibited higher anti-proliferative potency against H157. The results indicate that HECI, inhibiting chymotryptic-like activity of proteasome, could provide new insights in treatment of lung cancer.

## Introduction

Serine protease inhibitors are important in many crucial physiological processes within the human body^1^. Among three serine inhibitor families, the Bowman-Birk inhibitors (BBI) found in plants and amphibians, have been studied because of their potent protease inhibition. There are two groups of BBIs - one consists of large proteins which have more than one reactive site^2^ and the other consists of short looped peptides normally containing less than 20 residues with higher specific activity^3^.

Most amphibian-derived BBI peptides have been isolated from skin secretions of frogs from the family Ranidae, including *Lithobates pipiens* (pLR), *Lithobates sevosus* (pYR), *Odorrana versabilis* (HV-BBI), *Odorrana hejiangensis* (HJTI), *Hylarana latouchii* (pLR-HL) and *Odorrana schmackeri* (OSTI)^4–9^. These BBI peptides contain 17 to 18 amino acid residues, possess a common conserved internal sequence motif -WTKSXPPXP- and a single intramolecular disulphide bond which presents the conserved reactive loop^10^. They possess inhibitory activity with Ki values in the micromolar range^5–7,9,11^. Also, several BBI peptides are also highly potent tryptase inhibitors^6^ and others play important roles in the regulation of immune responses^4,12^.

Some BBI proteins such as the MSTI from snail medic seeds, BTCI from Black-eyed pea and a trypsin inhibitor from Hokkaido large black soybeans have anti-proliferative effects on tumour cells^13–15^. Several reports suggested that the anti-carcinogenic functions of serine protease inhibitors are probably related to their chymotrypsin-like and trypsin-like inhibitions on 20 S proteasome^15–17^. The inhibition of 20 S proteasome may suppress the proliferation of cancer cells through preventing the degradation of pro-apoptotic factors and inhibiting the activation of NFκB^18^.

Here, the discovery of the first chymotrypsin inhibitory BBI peptide from an amphibian skin secretion, is described from the Asian green frog, *Hylarana erythraea*. The peptide was isolated by RP-HPLC fractionation by nature of its chymotrypsin inhibitory activity determined by preliminary activity screening. The amino acid sequence was determined as TVLRGCWTFSFPPKPCI-amide, by Edman degradation and further confirmed by MS/MS fragmentation. An analogue (K^9^-HECI, TVLRGCWTKSFPPKPCI-amide) which was used for BBI inhibitor P1 position analysis, was synthesized in parallel with HECI.

## Results

### Identification and structural analysis of HECI

**Figure 1:**
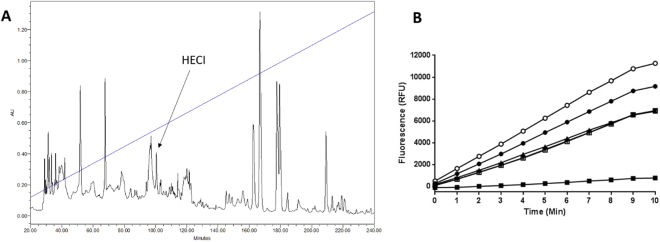
Identification and structural analysis of HECI. (**A**) RP-HPLC chromatogram of *Hylarana erythraea* skin secretion. The linear gradient of buffer B (0.05/19.95/80.0 (v/v/v) TFA/water/acetonitrile) is indicated in blue line. The arrow indicates the retention time of HECI, about 34% acetonitrile. (**B**) Chymotrypsin inhibition activity screening of HPLC fractions 98–102 min (○ 98 min, ● 99 min, ■ 100 min, □ 101 min, ▲ 102 min).

The skin secretion of *Hylarana erythraea* was fractionated by RP-HPLC over 240 min in the gradient from 0% to 100% buffer B (0.05/19.95/80.0 (v/v/v) TFA/water/acetonitrile) (Fig. 1A). Each fraction was reconstituted in PBS and subjected to the chymotrypsin inhibition assay and fraction # 100 was found to exhibit significant chymotrypsin inhibitory activity (Fig. 1B). The MS full-scan analysis of this fraction revealed the presence of a single molecule with a molecular mass of 1949.6 Da. Subsequent Edman degradation of this peptide found it to be composed of 17 amino acid residues - TVLRGCWTFSFPPKPCI - with a C-terminal amidation. MS/MS fragmentation confirmed the sequence as: TVLRGCWTFSFPPKPCI-NH_2_ (Table 1), and this novel BBI peptide was named *Hylarana erythraea* chymotrypsin inhibitor (HECI).

**Table 1:** MS/MS fragmentation sequencing data of HECI.

#1	b(1+)	b(2+)	Seq.	y(1+)	y(2+)	#2
1	102.055	51.531	T			17
2	201.123	101.065	V	1849.961	925.484	16
3	314.207	157.607	L	1750.892	875.950	15
4	470.309	235.658	R	1637.808	819.408	14
5	527.330	264.169	G	1481.707	741.357	13
6	630.339	315.673	C	1424.685	712.846	12
7	816.419	408.713	W	1321.676	661.342	11
8	917.466	459.237	T	1135.597	568.302	10
9	1064.535	532.771	F	1034.549	517.778	9
10	1151.567	576.287	S	887.481	444.244	8
11	1298.635	649.821	F	800.449	400.728	7
12	1395.688	698.348	P	653.380	327.194	6
13	1492.741	746.874	P	556.328	278.668	5
14	1620.836	810.921	K	459.275	230.141	4
15	1717.888	859.448	P	331.180	166.094	3
16	1820.898	910.952	C	234.127	117.567	2
17	—	—	I-Amidated	131.118	66.063	1

Through CID collision, the parent ion was fragmented into singly/doubly charged ions. The observed m/z ratios of b-ions y-ions are single-underlined.

### Molecular cloning of the HECI precursor-encoding cDNA from a skin secretion-derived cDNA library of Hylarana erythraea

**Figure 2:**
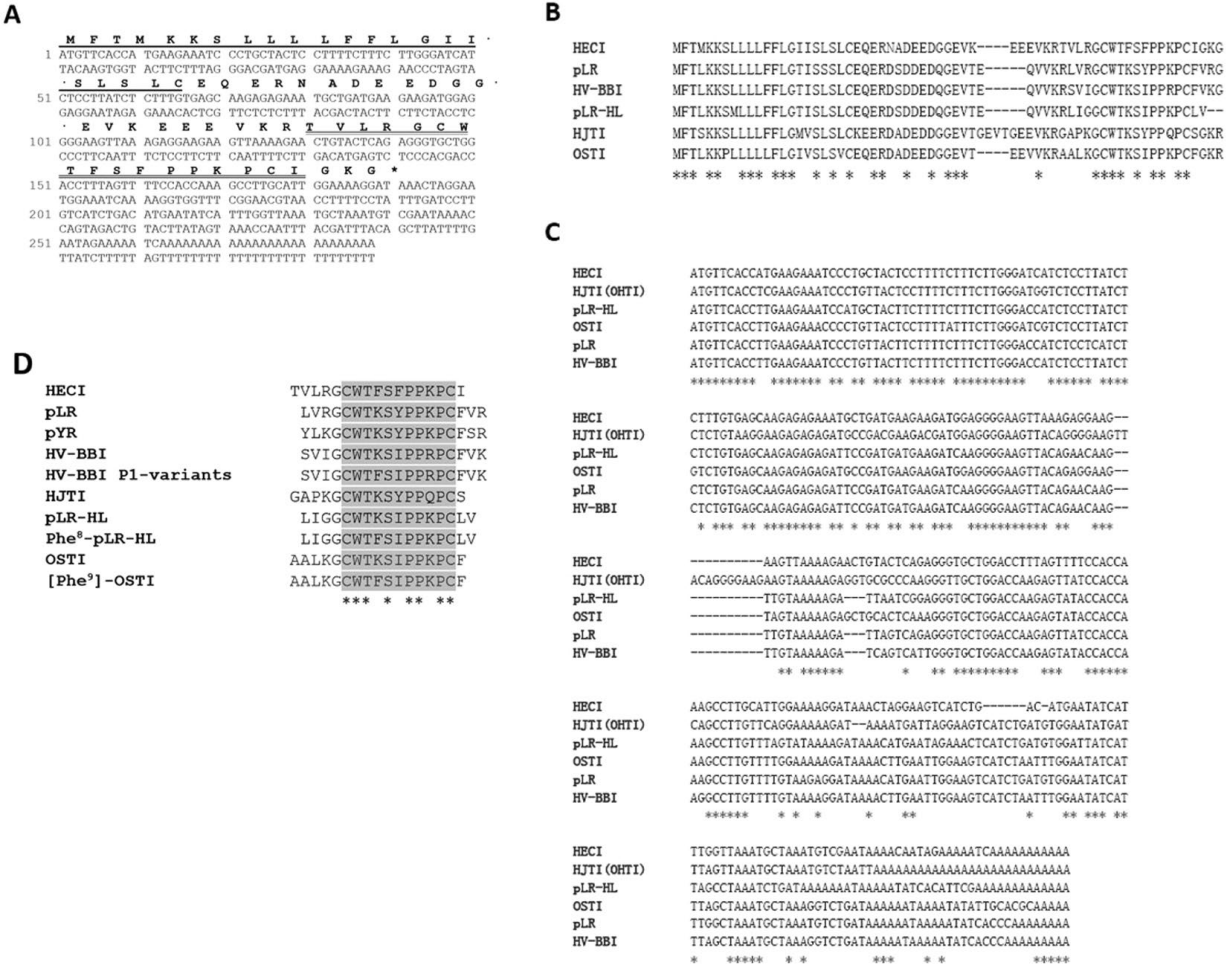
Molecular cloning of HECI precursor cDNA and sequence alignments. (**A**) The nucleotide and translated open reading frame amino acid sequence of the cDNA encoding the biosynthetic precursor. The putative signal peptide is single underlined and the mature peptide is double underlined. (**B**) The alignment of prepropeptides of amphibian BBI peptides. (**C**) The alignment of prepropeptide encoding cDNAs of amphibian BBI peptides. (**D**) The alignment of amphibian skin-derived BBI mature peptides with the reactive site loop of peptides in bold typeface. Identical amino acids and nucleotides are indicated by asterisks.

A single transcript encoding the biosynthetic precursor of the novel peptide named, HECI, was repeatedly cloned from the library. From this, both the nucleotide and translated open reading frame amino acid sequence of the HECI precursor were successfully determined (Fig. 2A). The biosynthetic precursor of HECI consisted of 63 amino acids organized as a putative signal peptide domain of 22 residues, which was followed by a 21-mer acidic amino acid residue-rich “spacer” peptide. The mature peptide sequence followed a typical processing site (-KR-) and ended in a Gly residue, which is a classical amide donor for C-terminal amidation. The alignments of the prepropeptides and the cDNAs of amphibian skin-derived BBI peptides showed that they exhibited high degrees of similarity. The signal peptide domain and the inhibitory motif within the mature peptides, were both highly-conserved (Fig. 2B and C). The loop structure of the HECI mature peptide possessed the same motif, WTKSXPPXP, as other skin-derived BBI peptides. The distinctive structural feature of HECI is that two Phe residues occupy the positions at P1 and P2’ (Fig. 2D)^4–7,9,12^. The cDNA sequence of the HECI precursor has been deposited in the Nucleotide Sequence Database under the accession code, MF668238.

### Syntheses of HECI and its K^9^-substituted analogue

**Figure 3:**
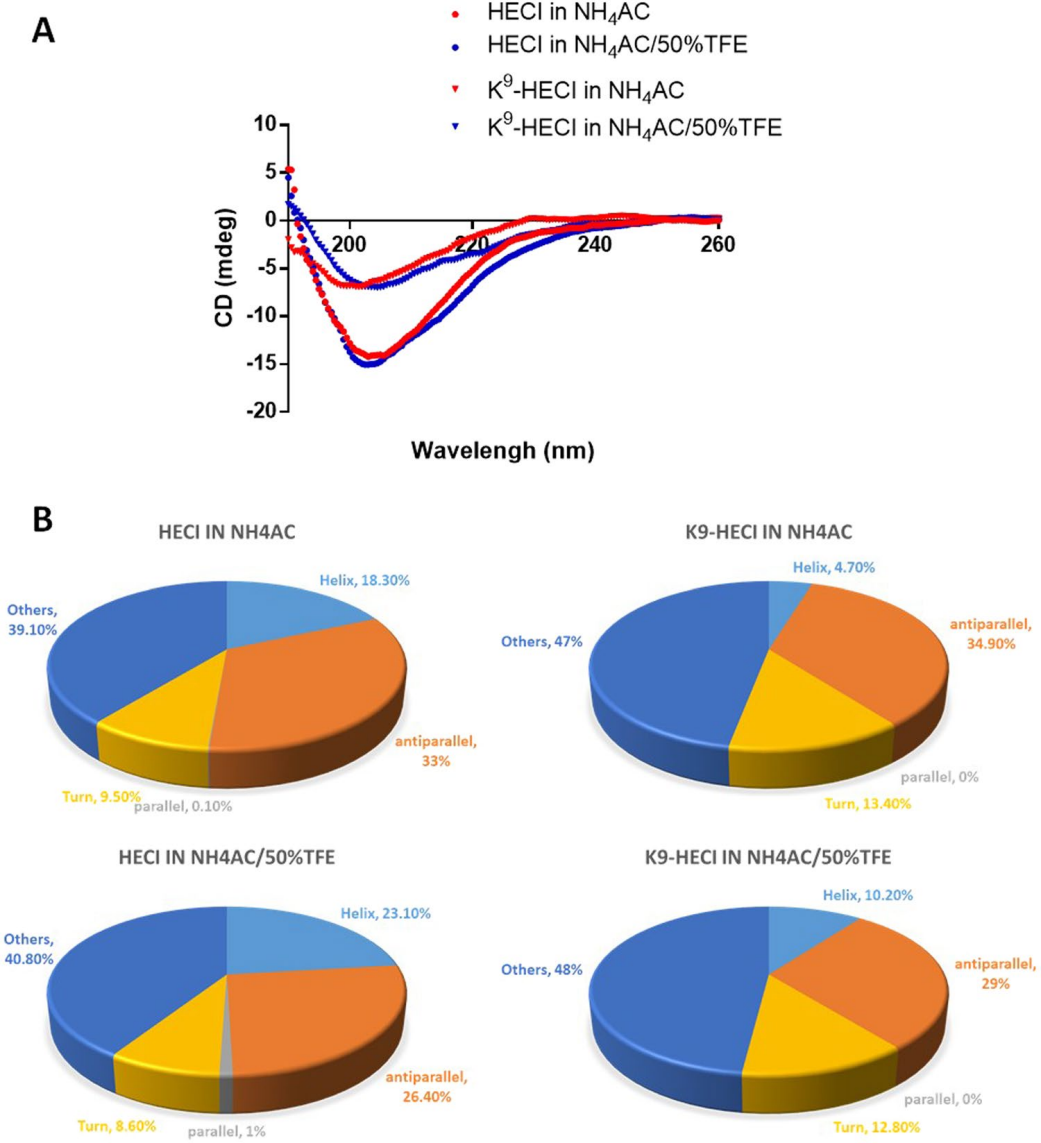
Secondary structure analyses of HECI and K^9^-HECI. (**A**) The CD spectra of HECI (dot) and K^9^-HECI (triangle) were measured in aqueous NH_4_AC buffer (red colour) and membrane-mimic NH_4_AC/50%TFE buffer (blue colour), respectively. (**B**) The proportion of different secondary structures domain were predicted and calculated using the online software, BeStSel^35^.

HECI and K^9^-HECI were successfully synthesized with a disulphide bond formed between C^6^ and C^16^. Both peptides formed similar secondary structures in 50% TFE/10 mM ammonium acetate buffer and 10 mM ammonium acetate buffer (Fig. 3A). The percentage of each secondary structure type of each peptide in each solvent environment, was calculated and results are shown in Fig. 3B.

### Trypsin/Chymotrypsin/20 S Proteasome inhibitory activity of HECI and its K^9^-substituted analogue

**Figure 4:**
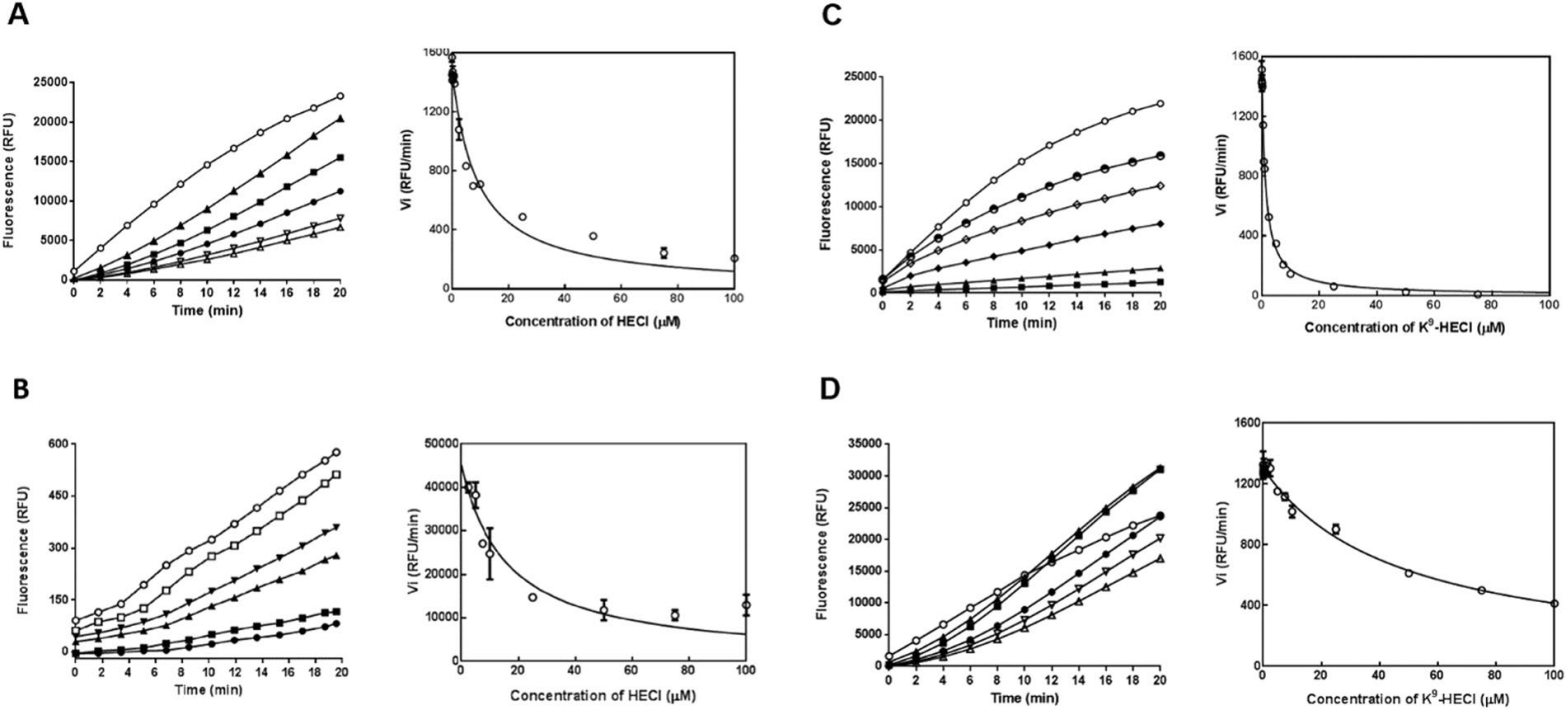
Inhibitory activity of HECI and the Lys-substituted analogue on trypsin, chymotrypsin and the 20 S proteasome. (**A**) Progress curves and corresponding Morrison plot for chymotrypsin proteolysis in the presence of different concentrations of HECI. (**B**) Progress curves and corresponding Morrison plot for human proteasome 20 S proteolysis in the presence of different concentrations of HECI. (**C**) Progress curves and corresponding Morrison plot for trypsin proteolysis in the presence of different concentrations of K^9^-HECI. (**D**) Progress curves and corresponding Morrison plot for chymotrypsin proteolysis in the presence of different concentrations of K^9^-HECI. (△ 100 μM, ▽ 75 μM, ● 50 μM, ■ 25 μM, ▲ 10 μM, ▼ 7.5 μM, □ 5 μM, ♦ 2.5 μM, ◇ 1 μM, ◓ 0.25 μM, ○ 0 μM).

HECI exhibited inhibition activity against chymotrypsin with a Ki value of 3.92 ± 0.34 μM (Fig. 4A). It also inhibited the chymotrypsin-like activity of the human 20 S proteasome at a similar concentration with a Ki of 8.55 ± 1.84 μM (Fig. 4B). K^9^-HECI showed inhibitory effects against trypsin with a Ki value of 0.79 ± 0.04 μM (Fig. 4C). Unexpectedly, the chymotrypsin inhibitory activity of K^9^-HECI remained. The Ki value was determined to be 13.88 ± 1.09 μM, which was lower than that of HECI (Fig. 4D).

### Cell viability assays of HECI and its K^9^-substituted analogue on cancer cells, normal cells and erythrocytes

**Figure 5:**
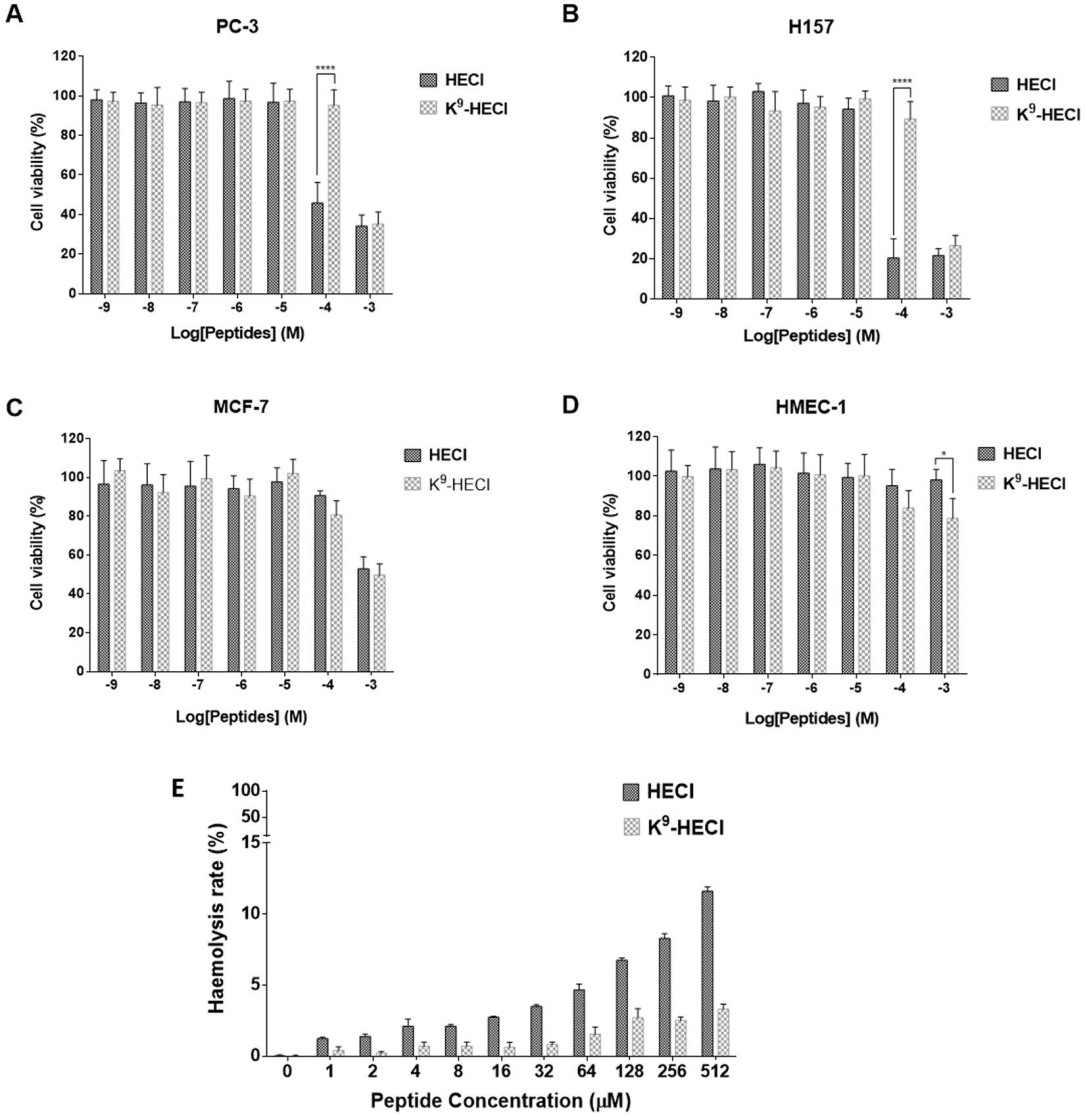
Effects of HECI and the Lys-substituted analogue on cancer cell lines, a normal cell line and erythrocytes. Cell viability of cancer cell line (**A**) PC-3, (**B**) H157, (**C**) MCF-7 and (**D**) the normal cell line HMEC-1 at different concentrations of HECI (dark grey) and K^9^-HECI (light grey). Statistical significance of difference was analysed by two-way ANOVA (*p < 0.05, ****p < 0.0001). (**E**) Haemolysis rates of HECI and K^9^-HECI on erythrocytes after being incubated for 4 h. The sample for the 0 μM control group was PBS only.

Both HECI and K^9^-HECI were subjected to MTT anti-proliferation assays using PC-3, H157 and MCF-7 cancer cell lines. Besides HECI was able to inhibit the growth of PC-3 and H157 at 100 μM, both peptides inhibited the growth of all cancer cells at 1 mM (Fig. 5A and B). However, both HECI and K^9^-HECI exhibited low degree of inhibition on the normal cell line, HMEC-1 (Fig. 5C). Moreover, both peptides showed low degree of haemolytic activity horse erythrocytes (Fig. 5D).

## Discussion

In recent decades, short BBI peptides have been found in amphibian skin secretions, which possess potent trypsin inhibitory activity. However, to date, there have been no natural amphibian BBI peptides reported with activity against chymotrypsin. Here, we report the first example of a natural chymotrypsin inhibitory BBI peptide, named HECI, from the skin secretion of the Asian green frog, *Hylarana erythraea*.

The signal peptide sequence and cDNA sequence of the biosynthetic precursor of this natural peptide are similar to those encoding other BBI peptides from amphibian skin secretions. However, the amino acids at P1 and P2′ positions of HECI are both Phe residues, which is consistent with observed chymotrypsin inhibition in this peptide and in other such BBIs^19^. As expected, HECI showed moderate chymotrypsin inhibitory activity similar to that observed for P1 Phe-substituted analogues of amphibian skin-derived trypsin inhibitory BBI peptides^5–7,9,11^. Hence, the peptide analogue K^9^-HECI was designed and synthesized to determine if trypsin inhibition activity could be reverse engineered. Interestingly, K^9^-HECI was found to have both trypsin and chymotrypsin inhibitory activities, therefore we deduced that the P2′ position was also a key position for inhibition specificity of short BBI peptides. This hypothesis differs with respect to the function of P2′ sites in large BBI proteins, which are related to the peptide hydrolysis rate^20^.

Several previous reports have shown that chymotrypsin inhibitors generally also have partial inhibitory activities on the proteasome^8^. Thus, a proteasome inhibition test on HECI was performed. The result showed that HECI had moderate inhibition on the chymotrypsin-like active site of the human 20 S proteasome. Indeed, the highest concentration of HECI even showed a more effective inhibition of the 20 S proteasome than it did on chymotrypsin. Additionally, the time of HECI inhibition of the 20 S proteasome was longer than that observed for chymotrypsin, which might be explained by the higher affinity of HECI for the 20 S proteasome. As we known, 20 S proteasome consists of 28 subunits, three of which conduct the tryptic (β2), chymotryptic (β5) and caspase-like (β1) activities, respectively^21^. Whilst, the catalytic nucleophile of proteasome subunits is the highly-conserved N-terminal threonine^22^. Inhibition of proteasome activities and subunit-specific amino-terminal threonine modification by lactacystin^23^, differing from the conventional serine in the active site, which may explain the different affinity of HECI between chymotrypsin and 20 S proteasome β5 subunit. As the 20 S proteasome plays a critical role in regulating many processes in the cell that are important for cancer cell growth and survive, and it mainly involves the chymotrypsin-like activity^24^. We therefore assumed that HECI might have anticancer properties, which indeed were confirmed in this study.

The major hypothesis of anti-cancer activity of peptides possessing chymotrypsin inhibition is that the peptide might be translocated into the cell and react with the related intracellular enzymes like the 20 S proteasome. Also, recent research support the idea that the anti-carcinogenic functions of some BBIs might be related to their proteasome inhibition^25^. Although both peptides inhibited the growth of three cancer cells up to the highest concentration, HECI shows more potent activity against the non-small cell lung cancer, H157 than the others. Indeed, proteasome inhibitor has been proved to inhibit a range of cancer cell lines but much more potent against H460, another non-small cell lung cancer^18^, which might be related to the NF-κB-mediated antiapoptotic pathway in non-small cell lung cancer^26^ that decreased the level of Bcl-2 to directly associate with apoptosis^27^. Interestingly, both peptides showed similar degree of inhibition at 1 mM on each cancer cell lines. In this study, we only investigated the chymotryptic-like activity of proteasome instead of all the catalytic sites, though the K^9^-HECI might be able to inhibit the tryptic-like β5 subunit. Previous study showed that plant-derived BBI, processing the inhibitory activity on those three subunits, inhibited the MCF-7 by means of apoptosis^15^, indicating that inhibition of all the subunits could contribute to the apoptosis-induced ability. Obviously, a lower anti-proliferation inhibitory activity of K^9^-HECI was observed that confirmed the chymotrypsin-like subunit produces the lead effect on anti-proliferation of cancer cells^15,28,29^. That the similar potency of both peptides on MCF-7 but the distinct activity on H157 and PC-3 also suggests different apoptosis pathways might be associated with proteasome inhibition. Additionally, considering that membrane transport is required of the peptide, more investigations would be needed to assess the transmembrane permeation capabilities of HECI.

Meanwhile, some amphibian skin derived BBIs exhibited antimicrobial activity, which was reported to insert into the hydrophobic core of membrane to form transmembrane pores^30^. The amphipathic peptide can increase the membrane permeability that results in the co-delivery of complex molecules from amphibian skin secretion^31^. It is possible that both peptides could compromise the cell membrane, resulting the inhibition of cell proliferation. Additionally, the CD spectra show that HECI has 2-fold greater helix content than K^9^-HECI in a membrane-mimic environment. When the HECI attaches to the cell membrane, it could be more efficient in interacting with the cell membrane than K^9^-HECI due to its higher hydrophobicity as a consequence of the highly hydrophobic region (-WTFSFPP-) of HECI^32,33^. When increasing the concentration to 1 mM, both peptides, losing specificity, showed similar potency against all cancer cells, which could be explained by the non-specific membrane permeabilization.

However, both HECI and K^9^-HECI showed only slight cytotoxicity on HMEC-1 and low degree of haemolysis of mammalian erythrocytes, which suggested that both proteasome inhibition and membrane permeabilization would occur in the antiproliferative activity on cancer cells, but the cell apoptosis through inhibition of proteasome might be predominant. Additionally, both peptides exhibited low degree of cytotoxicity on the normal cell line, indicating that it might be able to specifically target cancer cells, especially H157. The reason behind this is not clear so far, but an explanation of the low cytotoxicity on normal cells is that the expression level of proteasome inhibitor could be at a much lower level in normal cells, but higher in cancer cells^34^.

In summary, this is the first report of a chymotrypsin inhibitory BBI peptide in amphibian skin secretion, from the Asian frog, *Hylarana erythraea*. The peptide possesses inhibitory activities on chymotrypsin and the 20 S proteasome and its P1 Lys-substituted analogue showed potent trypsin inhibitory activity. HECI also showed more potent anticancer activity against PC-3 and H157 cell lines with low degree of cytotoxicity against normal cell lines. This study of HECI has provided a new insight into the possible clinical applications of skin-derived BBI peptides as well as their roles as potential candidates for natural drug development.

## Methods

### Acquisition of *Hylarana erythraea* skin secretion

The skin secretions of four *Hylarana erythraea* frogs were obtained by gentle electrical stimulation and hand massaging, which was described in detail previously (Tyler *et al*., 1992). Briefly, the moist frog dorsal skin was stimulated by 3~5 V, 100 Hz, AC. Then the electrode was moved slowly on the skin which was covered by glands for 10 s. Finally, the white secretion was washed off by deionized water and immediately snap frozen in liquid nitrogen. The samples were lyophilized for future analysis. The study was performed according to the guidelines in the UK Animal (Scientific Procedures) Act 1986, project license PPL 2694, issued by the Department of Health, Social Services and Public Safety, Northern Ireland. Procedures had been vetted by the IACUC of Queen’s University Belfast, and approved on 1st March, 2011.

### Initially fractionation of skin secretion

Five milligrams of lyophilized skin secretion were dissolved in 1 ml of deionized water containing 0.05% (v/v) trifluoroacetic acid (TFA, obtained from Sigma-Aldrich, St. Louis, MO, USA). The sample was subsequently centrifuged and the supernatant was further filtered through a 0.45 μm RC membrane (Phenomenex, Macclesfield, UK). The filtrate was injected into a reverse-phase HPLC system (UV Detector: Waters 2489; Binary HPLC Pump: Waters 1525; Autosampler: Waters 2707 (Waters Ireland, Dublin, Ireland); Column: Phenomenex Aeris Peptide, C18, 250 × 10.0 mm (Phenomenex, Macclesfield, UK), followed by elution with a gradient program from 0.05/99.95 (v/v) TFA/water (0 min) to 0.05/19.95/80.0 (v/v/v) TFA/water/acetonitrile in 240 min. The column effluent was monitored by UV absorbance at 214 nm, and fractions were collected automatically at 1 min intervals.

### Chymotrypsin inhibition assay screen

HPLC fractions were dried by Eppendorf Concentrator plus (Eppendorf, Hamburg, Germany) in the alcohol/vacuum mode and subsequently reconstituted using 20 μl of PBS and each was assayed in duplicate. A volume of 180 μl of substrate working solution containing 50 μM of Succinyl-Ala-Ala-Pro-Phe-AMC (Sigma-Aldrich, St. Louis, MO, USA), was added to each well in a black 96-well plate. 10 μl of each sample was added into the wells. Then 10 μl of chymotrypsin working solution (1 µg/ml) were added to each well. The rate of hydrolysis of the chymotrypsin substrate was monitored subsequently at wavelengths of 460 nm emission and 395 nm excitation, at 37 °C, by measuring the rate of increase of fluorescence in a FLUOstar OPTIMA multi-well plate reader (BMG Labtech, Ortenberg, Germany).

### Identification and structural analysis of HECI

Molecular mass analysis of the contents of the HPLC fraction exhibiting maximal chymotrypsin inhibitory activity was achieved by use of an LCQ-Fleet mass spectrometer (Thermo Fisher Scientific, San Jose, CA, USA). The major peptide within this fraction was subjected, without further purification, to automated Edman degradation using an Applied Biosystems Procise 491 microsequencer (Applied Biosystems, Foster City, CA, USA) in liquid phase delivery mode. Finally, the primary structure of the novel chymotrypsin inhibitory peptide was confirmed by MS/MS fragmentation sequencing. The detection was performed in positive ion mode. The ion capillary tube was heated to 320 °C and the spray voltage was set at 4.5 kV. The MS/MS spectrum was analysed using Thermo Scientific Proteome Dicoverer 1.0 software Sequest algorithm (Thermo Fisher Scientific, San Jose, CA, USA).

### Molecular cloning of HECI precursor-encoding cDNA from the skin secretion cDNA library

A further 5 mg of lyophilized secretion was dissolved in lysis/binding buffer of the Dynabeads® mRNA DIRECT™ Kit (Dynal Biotech Ltd., Bromborough, UK) which was used to isolate the mRNA from the secretion. The isolated mRNA was subjected to 5′- and 3′- rapid amplification of cDNA ends (RACE) procedures to obtain full-length peptide precursor nucleic acid sequence data by means of a BD SMART™ RACE cDNA Amplification Kit (BD Biosciences Clontech, Palo Alto, CA, USA), and was further subjected to the 3′-RACE procedures using a degenerate primer (5′- GGNTGYTGGACNTTYWSNTTY-3′, S = C + G; N = A + C + G + T; Y = T + C; W = A + T) that was designed to the internal amino acid sequence, -GCWTFSF-, of HECI. The 3′-RACE PCR products were purified (E.Z.N.A.® Cycle Pure Kit) (Omega Bio-Tek Inc., Norcross, GA, USA), and cloned using a pGEM^®^-T Easy Vector (Promega Corporation, Madison WI, USA). The nucleotide sequences of cloned cDNAs were obtained by an ABI3730 automated sequencer (Applied Biosystems, Foster City, CA, USA). Following the obtaining of the 3′-RACE product sequence, a specific antisense primer was designed to a site within the 3′ non-translated region. The 5′-RACE reaction was performed by using this specific antisense primer (5′-CCACATCAGATGACTTCCTAATCAT-3′) in conjunction with the UPM RACE primer. Generated PCR products were gel purified, cloned, and sequenced as described above.

### Synthesis of HECI and the Lys-substituted analogue

The natural HECI (TVLRGCWTFSFPPKPCI-NH_2_) and the Lys-substituted analogue (K^9^-HECI, TVLRGCWTKSFPPKPCI-NH_2_) were chemically synthesized using a solid-phase peptide synthesis approach to obtain sufficient samples for assaying bioactivities. Briefly, the Fmoc protection groups were removed by 20/80 (v/v) Piperidine/dimethylformamide (DMF, obtained from Fisher Scientific Ltd, Loughborough, UK) and the peptide bond was coupled in the presence of 2-(1H-benzotriazol-1-yl)-1,1,3,3-tetramethyluronium hexafluorophosphate (HBTU, obtained from Novabiochem®, Darmstadt, Germany), dissolving in 11/89 (v/v) N-methylmorpholine (NMM, obtained from Fisher Scientific Ltd, Loughborough, UK)/DMF. The process employed Rink amide resin and Fmoc amino acids (Novabiochem®, Darmstadt, Germany), and was performed in a Tribute® Peptide Synthesizer (Protein Technologies, Tucson, AZ, USA). The synthesized peptide was cleaved from the resin by a reaction containing 2/2/2/94 (v/v/v/v) 1,2-ethanedithiol/H_2_O/thioanisole/TFA. The peptide was washed using diethyl ether and dissolved in 0.05/99.95 (v/v) TFA/water. A final concentration of 0.01% of H_2_O_2_ was added to perform the formation of the disulphide bond.

### Determination of trypsin/chymotrypsin/human proteasome 20 S inhibitory activity of HECI and the Lys-substituted analog

Phe-Pro-Arg-AMC, Succinyl-Ala-Ala-Pro-Phe-AMC and Suc-Leu-Leu-Val-Tyr-AMC (Abcam, Cambridge, UK) were used as substrates for trypsin, chymotrypsin and human 20 S proteasome respectively. 10 μl trypsin/chymotrypsin working solution (1 µg/ml) were added to the wells of a micro-titre plate containing 180 μl substrate and various inhibitor solutions. 10 μl human 20 S proteasome (Biochem, Boston, MA, USA) working solution (7 nM) in 50 mM HEPES buffer containing 0.035% SDS, pH 7.5 was added to the well of a micro-titre plate containing 180 μl substrate solution and each of various inhibitor solution. Each determination was carried out in duplicate. The rate of hydrolysis of the trypsin/chymotrypsin substrate was monitored subsequently and continuously, at 37 °C, by measuring the rate of increase of fluorescence in a FLUOstar OPTIMA multi-well plate reader (BMG Labtech, Ortenberg, Germany). For the 20 S proteasome, the rate of hydrolysis of the substrate was monitored after a pre-incubation for 15 min, at 37 °C. The inhibition curves were plotted using Morrison equation, which is showed as follow:

1 $$Q=Ki\times (1+\frac{S}{Km})$$

2 $$y={V}_{0}\times (1-\frac{(Et+x+Q)-\sqrt{{(Et+x+Q)}^{2}-4\times Et\times x}}{2\times Et})$$

where, Et is the concentration of enzyme catalytic sites; S is the concentration of substrate; Km is the Michaelis-Menten constant; and V_0_ is the enzyme velocity with no inhibitor. Each assay was repeated three times.

### Secondary structure analyses of HECI and the Lys-substituted analogue through circular dichroism (CD)

The sample peptide solutions (50 μM) were prepared in a 1 mm high precision quartz cell (Hellma Analytics, Essex, UK) by 10 mM ammonium acetate and 50% TFE in 10 mM ammonium acetate buffer respectively. CD measurements were performed at room temperature by a JASCO J-815 CD spectrometer (Jasco, Essex, UK) across the wavelength range from 190 nm-260 nm. The scanning speed was 100 nm/min, the bandwidth was 1 nm and the data pitch was 0.5 nm. The CD spectra were further analysed using the online software, BeStSel^35^, and the following proportion of different secondary structures were predicted.

### Cell viability test of peptides on cell lines

The cancer cell line H157 (ATCC-CRL-5802, ATCC, Teddington, Middlesex, UK) and PC-3 (ATCC-CRL-1435, ATCC, Teddington, Middlesex, UK) were cultured in RPMI-1640 culture medium (Invitrogen, Paisley, UK) containing 10% FBS and 1% Penicillin-Streptomycin. MCF-7 (ATCC-HTB-22, ATCC, Teddington, Middlesex, UK) was cultured in DMEM culture medium (Invitrogen, Paisley, UK) containing 10% FBS and 1% Penicillin-Streptomycin. The normal cell line HMEC-1 (ATCC-CRL-3243, ATCC, Teddington, Middlesex, UK) was cultured in MCDB131 medium (Gibco, Paisley, UK) containing 10% FBS (foetal bovine serum, obtained from Sigma-Aldrich, St. Louis, MO, USA), 1% Penicillin-Streptomycin (Invitrogen, Paisley, UK), epidermal growth factor (10 ng/mL), hydrocortisone (1 μg/mL, obtained from Sigma-Aldrich, St. Louis, MO, USA) and glutamine (10 mM). Cells were seeded into a 96-well plate and incubated for 24 h (37 °C, 5% CO_2_) followed by starvation using serum-free medium. Sample solutions were loaded on this plate which was further incubated for 24 h. The 3-(4,5-dimethylthiazol-2-yl)-2,5-diphenyltetrazolium bromide (MTT) solution (5 mg/ml, 10 μl/well) was utilized to form the formazan after a 4 h incubation. Cell-produced-formazan was dissolved in DMSO (100 μl/well). The absorbance was recorded by using an ELx808^TM^ Absorbance Microplate Reader at 570 nm (BioTek Instruments, Inc., Winooski, VT, USA). All the peptide concentrations and controls had 5 replicates in single 96-well plate and three independent experiments were performed.

### Haemolysis activity test of peptides using horse erythrocytes

Completely washed horse erythrocyte (TCS Biosciences Ltd., Buckingham, UK) suspension was prepared using phosphate-buffered saline (PBS). A series of peptide solutions were incubated with a 2% suspension of red blood cells at final concentrations of 1–512 μM at 37 °C for 4 h. After the incubation, 200 μl of each supernatant was transferred into a 96-well plate and the absorbance at 550 nm was measured with a Synergy HT plate reader (BioTek Instruments, Inc., Winooski, VT, USA). The BioTek’s Gen5^TM^ software was used to analyse the result. (Biolise BioTek EL808, Winooski, VT, USA). All the peptide concentrations and controls had 5 replicates in single 96-well plate and three independent experiments were performed.

### Data availability

All data generated or analysed during this study are included in this published article.
